# The Impact of Metastasectomy on Survival Outcomes of Renal Cell Carcinoma: A 10-Year Single Center Experience

**DOI:** 10.3390/cancers15133332

**Published:** 2023-06-25

**Authors:** Mariaconsiglia Ferriero, Loris Cacciatore, Mario Ochoa, Riccardo Mastroianni, Gabriele Tuderti, Manuela Costantini, Umberto Anceschi, Leonardo Misuraca, Aldo Brassetti, Salvatore Guaglianone, Alfredo Maria Bove, Rocco Papalia, Michele Gallucci, Giuseppe Simone

**Affiliations:** 1 Department of Urology, IRCCS “Regina Elena” National Cancer Institute, 00144 Rome, Italy; mario.ochoa@gmail.com (M.O.); riccardo.mastroianni@ifo.it (R.M.); gabriele.tuderti@ifo.it (G.T.); manuela.costantini@ifo.it (M.C.); umberto.anceschi@ifo.it (U.A.); leonardo.misuraca@ifo.it (L.M.); aldo.brassetti@ifo.it (A.B.); salvatore.guaglianone@ifo.it (S.G.); alfredo.bove@ifo.it (A.M.B.); michele.gallucci50@gmail.com (M.G.); giuseppe.simone@ifo.it (G.S.); 2 Department of Urology, Fondazione Policlinico Universitario Campus Bio-Medico, 00128 Rome, Italy; loris.cacciatore@unicampus.it (L.C.); rocco.papalia@policlinicocampus.it (R.P.)

**Keywords:** renal cell carcinoma, oligometastatic RCC, metastasectomy, overall survival, non-evidence of disease

## Abstract

**Simple Summary:**

In the last years, metastasis-directed treatments of oligometastatic renal cell carcinoma (RCC) have been widely investigated. Metachronous solitary or oligometastasis from RCC are considered the ideal candidates for target treatments, allowing the achievement of “non-evidence of disease” status. To date, there have been no randomized clinical trials demonstrating the absolute survival benefits of surgical metastasectomy (MST) for oligo progression of RCC compared to systemic treatments. The role of complete MST on oncological outcomes, at the time of local or distant disease recurrence, remains poorly addressed. This is the first study presenting the advantage of minimally invasive MST on long-term (ten years) overall survival probability in patients who experienced oligoprogression of RCC treated at a high-volume center, compared to cases who received ST only.

**Abstract:**

Objectives: The role of surgical metastasectomy (MST) in solitary or oligometastasis from renal cell carcinoma (RCC) and its impact on survival outcomes remains poorly addressed. We evaluated the impact of MST on overall survival (OS) in patients with oligometastatic (m)RCC. Materials and methods: The institutional renal cancer prospective database was examined for cases treated with partial or radical nephrectomy who developed metastatic disease during follow-up. Patients with evidence of clinical metastasis at first diagnosis were excluded. Patients considered unfit for MST received systemic treatment (ST); all others received MST. The impact of MST vs. the ST only cohort was assessed with the Kaplan–Meier method. Age, gender, bilaterality, histology, AJCC stage of primary tumor, surgical margins, local vs. distant metastasis and MST were included in univariable and multivariable regression analyses to assess the predictors of OS. Results: Overall, at a median follow-up of 16 months after primary treatment, 168 patients with RCC developed asynchronous metastasis at the adrenal gland, lung, liver, spleen, peritoneal, renal fossa, bone, nodes, brain and thyroid gland. Nine patients unfit for any treatment were excluded. The site of metastasis was treated with surgical MST (77/159, 48.4%), with or without previous or subsequent ST, while 82/159 cases (51.2%) received ST only. The 2-year, 5-year and 10-year OS probabilities were 93.8%, 82.8% and 79.5%, respectively. After multivariable analysis, MST and the primary tumor AJCC stage were independent predictors of OS probabilities (*p* = 0.019 and *p* = 0.035, respectively). After Kaplan–Meier analysis, MST significantly improved OS probabilities versus patients receiving ST (*p* < 0.001). Limitations: The main drawbacks of our research were the small sample size from a single-tertiary referral institution, as well as the absent or different ST lines in the cohort of patients receiving MST. Conclusions: When an NED status is achievable, surgical MST of mRCC significantly impacts OS, delaying and not precluding further subsequent ST.

## 1. Introduction

Renal cell carcinoma (RCC) represents approximately 3% of all cancers and it is the most common variant histology of kidney cancer (approximately 90% of all kidney malignancies) [1]. With the introduction of minimally invasive surgical technology, an increased interest in nephron-sparing surgery, without precluding oncologic control of disease, represents the current urological challenge for the surgical management of RCC. Despite the early diagnosis and accurate surgical treatments, up to 35% of patients with localized tumors (5% for T1a, 15% for T1b, 35% for T2) and up to 47% of locally advanced disease (42% for T3, 47% for T4 tumors) can eventually develop local or distant recurrence [2] due to its immunogenic and biological features. In this context, the most common metastatic sites of RCC include the lungs (10–57%), followed by bone (16–27%), local (adrenal, retroperitoneal, renal fossa, 3–27%), the liver (1–12%) and the brain (2–4%) [3].

Nowadays, systemic treatment (ST), including tyrosine kinase inhibitors (TKIs) and immune checkpoint inhibitors, still plays a vital role in treatment for metastatic RCC (mRCC). Selective locoregional treatment of oligo progression is still an emerging concept in many tumors, such as prostate, colon, breast, etc. [4,5,6,7].

In recent years, novel metastasis-directed treatments of oligometastatic RCC have been introduced, such as stereotactic radiation and thermal ablation, showing adequate local control rates [8,9]. Brain and bone metastases are more often treated with stereotactic radiation therapy, while for the other sites, metastasectomy (MST) surgery remains the treatment of choice, with a consistent oncological benefit in terms of overall survival (OS), cancer-specific survival (CSS) and the delay of systemic therapy [1]. In the setting of the surgical management of mRCC, a meta-analysis by Zaid et al. reported that a complete MST was associated with a reduced risk of all-cause mortality compared to incomplete surgical MST (pooled aHR 2.37, 95% CI 2.03–2.87, *p* < 0.001), with low heterogeneity (I^2^ = 0%) [10]. On the other hand, a literature review by Matuszczak et al. [11] reported that every group of patients with mRCC with a limited metastatic burden can benefit from a MST surgical procedure as long as their global health status allows them to undergo this procedure. Therefore, the optimal therapy may be tailored individually; above all, for younger patients with good clinical condition and low comorbidities, a surgical approach should always be considered to achieve a more likely “non-evidence of disease” (NED) status.

To date, there have been no randomized clinical trials demonstrating the absolute survival benefits of surgical metastasectomy on oligo mRCC compared to ST; furthermore, the role of complete MST on oncological outcomes, at the time of local or systemic disease recurrence, remains poorly addressed. Metachronous solitary- or oligometastasis from RCC are considered the ideal candidates for target treatment, allowing the achievement of NED status and delaying the beginning or the shift to a more toxic treatment line. Thus, the aim of our study is to assess the impact of minimally invasive surgical MST on OS in patients who developed oligo progression of RCC treated in a tertiary referral center.

## 2. Materials and Methods

### 2.1. Patient Selection

The study was approved by the internal Institutional Review Board Statement and Ethics Committee. The Institutional renal cancer prospective database was examined for patients with diagnosis of RCC treated with either partial or radical nephrectomy who developed oligometastatic disease during follow-up. Oligometastatic disease was defined as the presence of three or fewer metastatic lesions after a histologic diagnosis of RCC. Metastatic sites were defined as *local* when occurring at the level of the renal fossa, the peritoneum and the skin (mainly at the level of the access ports); all the others were defined as *distant*. Cases with evidence of clinical metastases at first diagnosis were excluded. Patients with metastatic disease diagnosed within 3 months of initial diagnosis of cancer were excluded. Patients considered unfit for MST based on surgeons’ criteria (without an achievable non-evidence of disease status after surgery), received systemic treatment; all the others received MST. Metastasectomy was defined as complete resection of all metastatic sites. Cases deemed unfit for surgery due to comorbidities with an achievable NED status, were sent for radiation therapy or other ablative treatments but not included in the present analysis.

### 2.2. Data Collection and Follow-Up

Baseline demographic, clinical and pathologic data were prospectively collected. Postoperatively, lab exams (including electrolytes, renal and liver function), clinical examination, abdominal ultrasound and chest X-ray or CT scans were performed at six-month intervals during the first 2 years, followed by yearly evaluation thereafter. CT scans and MRI (when indicated) were the most common imaging modalities used to detect metastases and assess resectability.

### 2.3. Statistical Methods

Overall survival probabilities of the whole cohort were computed at 12, 24, 60 and 120 months after primary treatments. The impact of MST vs. ST only cohort (reference category) was assessed with the Kaplan–Meier method. Age, gender, bilaterality, histology, AJCC stage of primary tumor, positive surgical margins (PSM) and MST were included in univariable and multivariable Cox regression analyses to assess predictors of overall survival. All *p* < 0.05 were considered statistically significant. A descriptive statistic was performed with Statistical Package for Social Science (SPSS version 23, IBM, Chicago, IL, USA).

## 3. Results

**Figure 1:**
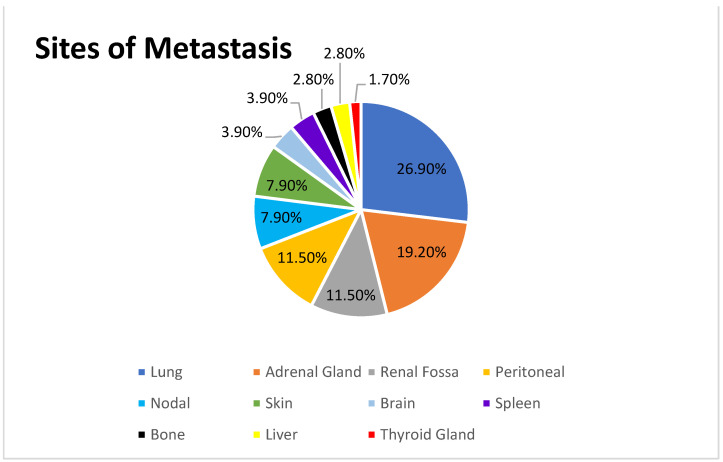
Sites of metastasis.

At a median follow-up of 16 months after primary treatment, 168 patients with RCC developed asynchronous metastasis at the adrenal gland, lung, liver, spleen, peritoneal, renal fossa, bone, nodes, brain and thyroid gland. The sites of metastasis are reported in Figure 1.

Nine patients were unfit for any treatment and were excluded. Clinical and pathological features of our cohort are reported in Table 1. The median age was 61 (IQR 47–70) years, with 111 male and 48 female patients. Primary treatments performed were radical nephrectomy in 108 patients (67.9%) and partial nephrectomy in 51 patients (32.1%). Concerning the final histological report after the first surgical treatment, the pT most represented was pT1b in 46 cases (28.9%) of the total cohort, while only 15 patients (9.5%) presented lymph node involvement at primary diagnosis and treatment. Only two patients (1.3%) presented positive surgical margins. In the present series, the predominant histology was clear cell RCC in 134 patients (84.3%), and the most represented AJCC stages were stage I and stage III in 69 patients (43.4%) and 61 patients (38.3%), respectively. The site of metastasis was treated with MST in 48.4% of cases (77/159 patients) with or without previous or subsequent ST, while 51.2% of cases (82/159 patients) received only ST. The two cohorts were homogenous for the main clinical features, such as age (*p* = 0.754), gender (*p* = 0.589), primary surgery (*p* = 0.294), histology (*p* = 0.697) and positive surgical margins (*p* = 0.952). Conversely, the two groups were different for AJCC stage and Fuhrman Grade (*p* = 0.016 and *p* = 0.018, respectively).

Among the ST used, the majority of patients received TKIs. Specifically, 46 patients (56.1%) received Sunitinib, while Pazopanib was administered to 21 patients (25.60%). The Clavien–Dindo high-grade (≥3) complication rate of MST performed by urologists (abdominal sites and skin) was 1.7%. The mean follow-up after secondary treatment was 22 months.

**Figure 2:**
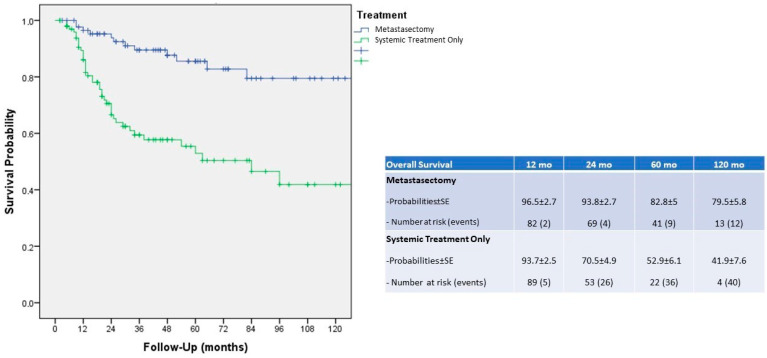
Kaplan–Meier analysis and overall survival probabilities after 1, 2, 5 and 10-year evaluation.

Upon univariable analysis, we found that a lower AJCC stage, metastasectomy and local metastasis were predictors of OS probability (*p* = 0.003, *p* < 0.001 and *p* = 0.031, respectively). In multivariable models, the primary tumor AJCC stage and MST were independent predictors of OS probabilities (*p* = 0.035 and <0.019, respectively) (Table 2). Upon Kaplan–Meier analysis, the 2-year, 5-year and 10-year overall survival probabilities were 93.8%, 82.8% and 79.5%, respectively (Figure 2). MST significantly improved OS probabilities versus patients receiving ST only (*p* < 0.001).

## 4. Discussion

Recently, there has been an unquestionable improvement in the postoperative outcomes (short hospitalization, reduced blood loss, low rates of complications) with minimally invasive surgical techniques, and an enlarged adoption of surgical treatment compared to the past in patients with advanced local or oligometastatic disease. Therefore, the role of surgery is now considered even in primary metastatic disease.

Recently, the findings of the CARMENA trial by Méjean et al. [12] showed comparable survival outcomes of patients receiving cytoreductive radical nephrectomy plus sunitinib versus sunitinib alone, but also a higher rate of severe adverse events in the sunitinib alone arm (42% vs. 33%, *p* = 0.04). This phenomenon could be due to the removal of the primary tumor, given the need for multiple lines of medical therapy, especially in intermediate- to poor-risk mRCC treated without surgical debulking. Nonetheless, the guidelines do not provide clear indications in metastatic disease as to who can benefit from an intensive oncological or surgical treatment with OS as the primary endpoint.

With this background, we evaluated the benefit of adding MST in patients experiencing oligometastases after partial or radical nephrectomy during a 10-year follow-up, compared to patients treated with ST only in our tertiary-care center. In the present series, clear cell RCC was the predominant histology (84.3%), in line with other previous studies (86.4–93.8%) [13,14]. Lung resection was the most common metastasectomy performed in many series (29.4–39.2%) [14,15]; similarly, in our study, the lung resection rate was prevalent (26.9%).

Dragomir et al. [14] and Samsel et al. [16] reported adrenal metastasectomy for mRCC in 16% and 14% of cases, respectively. In our series, adrenal gland resection cases were comparable to those reported in the literature (19.2%), being the second site of MST. Finally, the rates of peritoneal and renal fossa resections reported in the literature were 0.8–3.6% [17,18] and 3.74%, respectively [18]. Conversely, we performed a higher rate of metastasectomies for local and peritoneal recurrence (both 11.5%), compared to the previous findings. A meticulous follow-up could explain a more accurate detection of recurrence and a prompt identification of cases best suited for the further control of recurrent disease.

Few data are available in the literature concerning the safety of MST. Meyer et al. [19] reported, in a multicentric experience of 1102 cases of metastasectomies, a high-grade (Clavien III–V) complication rate of 27.5% in cases of MST performed with any approach. However, in this multicentric experience, the complication rate after locoregional treatment is missing, leaving the evaluation of MST safety to be further investigated. In the present study, we reported only the available complication rate of minimally invasive metastasectomies (mostly performed by a urologist), such as adrenalectomy, skin, peritoneal and renal fossa resections, while other information of MST performed in extra-abdominal sites and data concerning adverse events in patients treated with ST were not available in the present series. Published experiences of MST performed with an open approach showed a significant rate of high-grade Clavien–Dindo complications (ranging between 14.7% and 25%) [17,20]. A comparable rate of high-grade (Clavien III–IV) complications after laparoscopic or robotic metastasectomy was found, compared with those reported in the other series (1.7% vs. 1.6%) [21]. Even if mortality rate described in literature ranged between 2–2.4% [17,19], in our series we did not report any postoperative mortality complication (Clavien V complication rate: 0%). Our data show that minimally invasive techniques are guaranteed to achieve an acceptable control of oligometastatic disease with negligible impact on patient safety.

Concerning survival outcomes, Lyon et al. [22] showed that 2-year OS after MST was greater than the no MST group (84% vs. 54%, *p* < 0.001, respectively); moreover, Dragomir et al. [14] reported an improved 5-year OS after MST compared with those patients who did not receive MST (63.2% vs. 51.4%, *p* = 0.0001, respectively). Similarly, in our series, patients receiving MST displayed improved OS probabilities, compared to cases treated with ST only, even at a long-term follow-up (93.8%, 82.8% and 79.5% versus 70.5%, 52.9% and 41.9%, respectively for 2-year, 5-year and 10-year OS probabilities, *p* < 0.001).

In our series, the two groups, MST and ST only, were not homogenous for stage and grade of the primary tumor. We reported a higher rate of pT1a and pT1b lesions in those who developed metastases, compared to pT2a and pT2b tumors. This phenomenon could be explained by a simple epidemiological incidence, early diagnosis or higher metastatic potential of small renal masses. As a tertiary referral center for robotic partial nephrectomy, we perform a nephron-sparing surgery whenever feasible, even in challenging cases such as cT2, totally endophytic or purely hilar tumors [23,24]. With hematuria being the only indication of radical nephrectomy (regardless of the tumor size), our findings may be justified by the biologic activity of disease.

Similarly, Sharp et al. showed a stage migration to metastatic RCC for a higher proportion of patients presenting with cT1-2 disease, as opposed to cT3-4. Additionally, they observed that there is a possible increase in cT1 tumors with metastatic potential, presenting in 1 in 4 metastatic patients [25]. However, even in our series, the primary tumor AJCC stage remains one of the most essential predictors of oncological outcomes, guiding the selection of the best treatment choice for patients with RCC.

Previous studies identified several prognostic models predicting oncological outcomes of mRCC [26,27,28,29,30]. Concerning histological subtypes, a Leibovich score included a prognostic stratification for each of the three most common RCC subtypes, showing the ccRCC variant as the strongest predictor (C-index 0.83 versus 0.77 and 0.78 for ccRCC, papillary RCC and chromophobe RCC, respectively) [26]. Concerning bilaterality, Kim et al. reported an improved 5-year recurrence free survival probability of unilateral disease versus the bilateral RCC group (94.3% vs. 82.6%, *p* = 0.045) [31]. However, in the present series, ccRCC variant histology and bilateral disease did not impact on OS probability upon univariable analysis (*p* = 0.052 and *p* = 0.471, respectively). The positive surgical margin rate is considered an independent predictor of disease recurrence after nephron-sparing surgery for RCC (*p* = 0.013) [32], while the impact of PSM on OS probabilities is negligible in the literature [33] as well as in the present study (univariable Cox *p* = 0.953). Concerning metastatic sites, Abdel-Rahman [34] evaluated the clinical and prognostic value of metastatic sites in mRCC; the author reported that patients with liver metastases of RCC have worse outcomes and worst OS compared to cases who developed other sites of metastases. In the present series, a local site was found to be a predictor of OS probability (*p* = 0.031) after univariable analysis, while after multivariable analysis, only the primary AJCC stage and MST were predictors of OS probabilities, *p* = 0.035 and *p* = 0.019, respectively. Therefore, local sites were not associated with improved overall survival compared to distant sites of recurrence.

In the oligo-recurrent scenario, the only strong recommendation provided by the guidelines is to not offer tyrosine kinase inhibitor treatment to mRCC patients after MST and no evidence of disease [1]. Systemic treatments are often associated with adverse events, such as hypertension, palmar-plantar erythrodysesthesia, thrombocytopenia, asthenia, diarrhea, an increase in lipase enzymes, etc., being significant (Grade 3–4 according to CTCAE-5 classification) in 63% of cases and leading to treatment discontinuation in 12% [35]. Minimally invasive MST, related with a low complication rate, can be considered a safe treatment option in the case of oligo progression of disease. Our results emphasize the importance of surgical treatment of mRCC recurrence and encourage MST when an NED status is feasible, providing a long-term OS advantage towards patients receiving a more toxic ST.

However, our study is not devoid of limitations. The single-tertiary referral center analysis, the small sample size, as well as selection bias due to absent or different ST lines in the cohort of patients receiving MST, can be considered the main drawbacks of the study. Moreover, patients fit for MST were selected according to surgeon discretion, after an accurate anesthesiologic evaluation. Thus, we acknowledge that survival outcomes may be influenced by confounding factors as well as the difference between the two cohorts for the stage and grade of primary tumors. Moreover, the time of NED status after MST is not available; therefore, the survival analysis is based on OS probability.

Centralization of care of mRCC based on multidisciplinary shared decision making remains crucial when managing targeted treatments. Marchioni et al. [36] highlighted that the choice to perform MST in patients with oligometastasis should be based on a careful evaluation of patient health status and tumor characteristics (AJCC stage of native tumor) by experienced surgical and clinical teams, in order to identify the best candidates for MST treatment, for which the potential oncological benefits outweigh the perioperative risks.

This is the first study showing the advantage of MST on long-term OS probability in oligometastatic patients treated at a high-volume center, compared to those treated with systemic therapy only.

## 5. Conclusions

Metastasectomy can be considered an effective treatment option in selected patients after discussion in a multidisciplinary context. A careful surveillance and adequate follow-up guarantees the early detection of disease recurrence when an NED status is achievable. Prospective and larger studies are needed to support our findings.

## Figures and Tables

**Table 1:** Clinical and pathological features of cohort.

Clinical Features	Median or N (IQR or %)
**Age (years)**	61 (47–70)
**Gender**	
F	48 (30.2)
M	111 (69.8)
**ECOG**	
**0**	148 (93.1)
**1**	11 (6.9)
**Bilateral disease**	5 (3.1)
**Primary Treatment**	
Radical Nephrectomy	108 (67.9)
Partial Nephrectomy	51 (32.1)
**pT**	
x	4 (2.5)
1a	25 (15.7)
1b	46 (28.9)
2a	18 (11.3)
2b	10 (6.3)
3a	34 (21.4)
3b	18 (11.3)
3c	2 (1.3)
4	2 (1.3)
**pN**	
0	144 (90.5)
1	6 (3.8)
2	8 (5.1)
3	1 (0.6)
**AJCC stage**	
I	69 (43.4)
II	27 (16.9)
III	61 (38.3)
IV	2 (1.3)
**Histology**	
ccRCC	134 (84.3)
Other than ccRCC	25 (15.7)
**Positive Surgical Margins Rate**	2 (1.3)
**Follow-up time (months)**	16 (9–42)
**Treatment**	
MST	77 (48.4)
ST	82 (51.2)

**Table 2:** Cox regression analysis for overall survival.

COX Regression Analysis for Overall Survival								
	Univariable				Multivariable			
Variable	*p*	OR	95% CIs		p	OR	95% CIs	
			Low	High			Low	High
**Age**	0.838	1.00	0.96	1.05	-	-	-	-
**Gender**	0.647	1.07	0.79	1.45	-	-	-	-
**Bilaterality**	0.471	2.07	0.28	14.23	**-**	-	-	-
**ccRCC histology**	0.052	1.87	0.99	3.49	**-**	-	-	-
**AJCC stage (I ref.)**	**0.003**				**0.035**			
**II vs. I**	**0.138**	1.95	0.81	4.71	**0.146**	1.93	0.79	4.68
**III vs. II**	**0.001**	3.47	1.71	7.03	**0.036**	2.31	1.06	5.07
**IV vs. I**	**0.016**	6.52	1.42	30.01	**0.018**	13.22	1.56	111.77
**Positive Surgical Margins**	0.953	1.02	0.51	2.06	-	-	-	-
**Metastasectomy**	**<0.001**	3.89	2.07	7.32	**0.** **019**	2.77	1.18	6.47
**Local Metastasis (renal fossa, peritoneal, skin)**	**0.031**	4.80	1.16	19.91	**0.257**	2.50	0.51	12.23

## Data Availability

The data presented in this study are available if requested.
